# Supplementary material for: Different Expression of Mitochondrial and Endoplasmic Reticulum Stress Genes in Epicardial Adipose Tissue Depends on Coronary Atherosclerosis
Source: Int J Mol Sci. 2021 Apr 26;22(9):4538. doi: 10.3390/ijms22094538 (PMC8123607; doi:10.3390/ijms22094538)
Supplement: Supplementary file 1 [file ijms-22-04538-s001.zip › ijms-1179760-supplementary.pdf]

Supplementary table 1: **Mitochondrial genes assessed in the study** (Mt – mitochondrial)

Mitochondrial gene	DNA location	Mt complex location	Mt location
Citrate synthase (CS)	Nuclear DNA	Krebs cycle	Matrix
NADH dehydrogenase subunit (NDUFA12)	Mt DNA	NADH-coenzymeQ_{10} reductase (Complex I)	Inner membrane
Mitochondrially encoded NADH dehydrogenase 5 (MT-ND5)	Mt DNA	NADH-coenzymeQ_{10} reductase (Complex I)	Inner membrane
Succinate dehydrogenase complex flavoprotein subunit A (SDHA)	Mt DNA	Succinate-ubiquinone oxidoreductase (complex II)	Inner membrane
Cytochrome c oxidase subunit 4/1 (COX4/1)	Mt DNA	Cytochrome c oxidase (complex IV)	Inner membrane
Cytochrom c 1 (CYC1)	Mt DNA	Ubichinol cytochrome c reductase (complex III)	Inner membrane

Supplementary table 2: **Endoplasmatic reticulum stress genes assessed in the study**

ER gene	Location	Protein function
ATF4 (Activating transcription factor 4)	Target to the nucleus, chromosome 22:39.52 – 39.52 Mb	Stress-induced transcription factor, master regulator of the cellular stress response that promotes adaptation of cells to a limited availability of nutrients
ATF6 (Activating transcription factor 6)	ER membrane	Under stress conditions will get into the cell nucleus where it directly affects the expression of uncoupling protein response target genes
HSPA5 (Heat Shock Protein Family A (Hsp70) Member 5)	Lumen of the endoplasmic reticulum (ER)	Folding and assembly of proteins in the ER

DDIT3 (DNA damage-inducible transcript 3)	Member of the CCAAT/enhancer-binding protein – target to the nucleus, chromosome 12:57.52 – 57.52 Mb	Implicated in adipogenesis and erythropoiesis, is activated by endoplasmic reticulum stress, promotes apoptosis
